# The Modulatory Effects of Mesenchymal Stem Cells on Osteoclastogenesis

**DOI:** 10.1155/2016/1908365

**Published:** 2015-12-28

**Authors:** Wessam E. Sharaf-Eldin, Nourhan Abu-Shahba, Marwa Mahmoud, Nagwa El-Badri

**Affiliations:** ^1^Center of Excellence of Stem Cells and Regenerative Medicine, Zewail City of Science and Technology, Sheikh Zayed District, 6th of October City, Giza 12566, Egypt; ^2^Medical Molecular Genetics Department, Human Genetics and Genome Research Division, National Research Centre, Cairo 12411, Egypt; ^3^Stem Cells Research Group, Centre of Excellence for Advanced Sciences, National Research Centre, Cairo 12411, Egypt

## Abstract

The effect of mesenchymal stem cells (MSCs) on bone formation has been extensively demonstrated through several *in vitro* and *in vivo* studies. However, few studies addressed the effect of MSCs on osteoclastogenesis and bone resorption. Under physiological conditions, MSCs support osteoclastogenesis through producing the main osteoclastogenic cytokines, RANKL and M-CSF. However, during inflammation, MSCs suppress osteoclast formation and activity, partly via secretion of the key anti-osteoclastogenic factor, osteoprotegerin (OPG). *In vitro*, co-culture of MSCs with osteoclasts in the presence of high concentrations of osteoclast-inducing factors might reflect the *in vivo* inflammatory pathology and prompt MSCs to exert an osteoclastogenic suppressive effect. MSCs thus seem to have a dual effect, by stimulating or inhibiting osteoclastogenesis, depending on the inflammatory milieu. This effect of MSCs on osteoclast formation seems to mirror the effect of MSCs on other immune cells, and may be exploited for the therapeutic potential of MSCs in bone loss associated inflammatory diseases.

## 1. Introduction

Bone is a dynamic tissue that remodels constantly throughout the adult life. Bone remodeling involves degradation of old or damaged bone by osteoclasts (bone resorption) and subsequent deposition of new bone by osteoblasts (bone formation) [1]. Bone remodeling is physiologically required to maintain calcium homeostasis, in addition to repairing bone damage induced by mechanical stress or aging [2]. It is a tightly regulated process under the control of physical activities and numerous polypeptides (systemic hormones, cytokines, and locally released growth and differentiation factors) [3]. Perturbations in bone regulatory factors can lead to net loss or gain of bone mass. The rate of bone remodeling with enhanced bone resorption increases in a variety of skeletal disorders such as postmenopausal osteoporosis, periodontal diseases, Paget's disease, rheumatoid arthritis, and lytic bone metastasis [4, 5].

Mesenchymal stem cells (MSCs) (also referred to as mesenchymal or multipotent stromal cells) are non-hematopoietic precursors. They were initially isolated from bone marrow (BM) (BM-MSCs) by Friedenstein and colleagues, as stromal adherent, fibroblast-like cells that have the potential to differentiate into mesodermal derivatives (osteoblasts, adipocytes, and chondrocytes)* in vitro* and regenerate heterotopic bone tissue when implanted* in vivo* [6]. MSCs have also been derived almost from all postnatal [7], fetal [8], and extraembryonic tissues [9]. Importantly, all the extraskeletal tissues in which MSCs exist do not contribute to skeletal development, homeostasis, or repair [10]. However, they have already shown a potent therapeutic effect on bone regeneration and bone metabolism upon local or systemic application [11, 12].

Although MSCs can be identified by common phenotypic characteristics, no specific markers for MSCs have been defined yet [13]. To unify MSC characteristics across different tissue types and various culture conditions, the International Society for Cellular Therapy (ISCT) has proposed minimal criteria to define adherent cultured cells as MSCs. These criteria include (1) plastic adherence when maintained in standard culture conditions; (2) the expression of CD105, CD73, and CD90 and lack of expression of CD45, CD34, CD14 or CD11b, CD79a or CD19, and HLA-DR surface markers; and (3)* in vitro* tri-lineage differentiation to adipogenic, chondrogenic, and osteogenic cells [14].

Over the past few years, the therapeutic potential of MSCs has been exploited at preclinical and clinical settings [15, 16]. This may be attributed to two main functional paradigms. The first relates the effective ability of MSCs to specific engraftment at the site of injury [17, 18] and tissue replacement via multipotency [19]. Tracking studies showed that intravenously infused MSCs in different disease models were entrapped in the lungs, and only a transient portion appeared in the damaged organs. However, functional improvement was observed in such models with poor or absent transdifferentiation [20, 21]. These studies and others attributed the regenerative potential of MSCs to the second proposed paradigm, in which MSCs exert beneficial effects on other cells via secretion of bioactive molecules (paracrine action). MSC paracrine factors can be antiapoptotic, mitotic, supportive for tissue resident progenitors, angiogenic, immunomodulating, or chemoattractant [22, 23].

The role of MSCs within BM stroma is not limited to their function as the progenitors of various types of mesodramal cells (osteoblasts, chondrocytes, adipocytes, and marrow stromal cells). MSCs have also been demonstrated to produce regulatory factors that affect osteoclast development and bone resorption. However, the effect of MSCs on osteoclastogenesis seems to be complex and dependent on the pathophysiological environment. In this review, the controversial effects of MSCs, especially those derived from BM, on the processes of osteoclastogenesis and bone resorption are discussed.

## 2. Osteoclasts and Osteoclastogenesis

**Figure 1:**
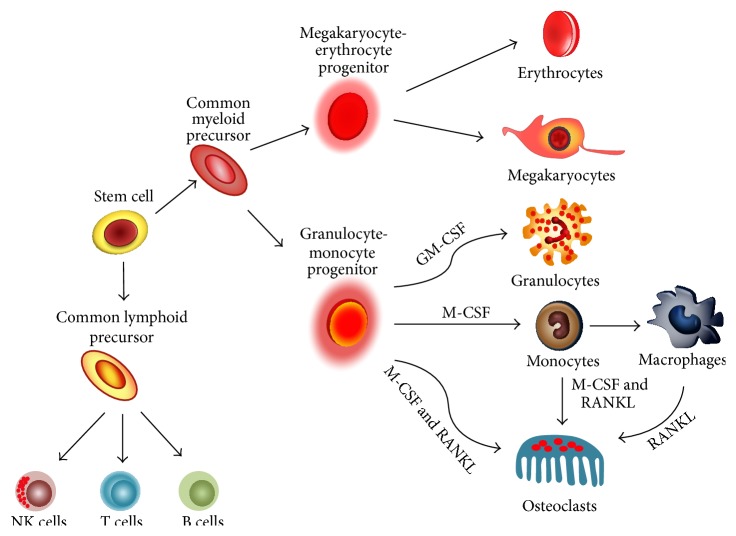
Origin of osteoclasts.

Osteoclasts are multinucleated, bone-resorbing cells. They develop efficient machinery for dissolving crystalline hydroxyapatite and degrading organic bone matrix rich in collagen fibers [24]. Osteoclasts originate from myeloid precursors, which arise from the bone marrow hematopoietic stem cells (BM-HSCs). They share a common origin with different immune cells such as megakaryocytes, granulocytes, monocytes, and macrophages [24–26]. Osteoclasts can be also derived from mature monocytes and macrophages when a suitable microenvironment is provided [27] (Figure 1). In addition to the common origin, osteoclasts play a phagocytic role in the bone, similar to that of macrophages in the immune system, and accordingly are called bone-specific macrophages [28]. Furthermore, osteoclasts function as immunomodulators in pathologic states, and via secreting various mediators, they participate in the pathogenesis of inflammatory bone loss [29]. Osteoclasts can thus be considered a member of the immune cells.

Osteoclast development (osteoclastogenesis) within a bone microenvironment is a multistep process. This sequential process is mainly under the control of an extensively investigated triad-system, which includes RANKL, RANK, and OPG [5]. Receptor activator for nuclear factor kappa B ligand (RANKL), alternatively named TNF-related activation induced cytokine (TRANCE) [30], osteoclast differentiation factor (ODF) [31], or osteoprotegerin ligand (OPGL) [32], is a member of the tumor necrosis factor (TNF) superfamily of cytokines [5]. RANKL, synthesized by mesenchymal cells, has been identified as the principal cytokine of the osteoclastic differentiation and activation during physiological bone remodeling [31]. RANKL signals through RANK expressed on osteoclasts and their progenitors [33, 34], inducing diverse cascades that mediate osteoclast development and activity [35]. To maintain normal bone homeostasis, RANKL signaling must be properly regulated. Osteoprotegerin (OPG) [36], also known as osteoclast inhibitory factor (OCIF) [32], is a non-signaling decoy receptor expressed by osteoblasts and other bone marrow stromal cells in response to anabolic agents such as estrogen and bone morphogenetic proteins (BMPs) [37]. OPG is a soluble member of the tumor necrosis factor receptor (TNFR) superfamily and it acts by disrupting the interaction between RANKL and RANK, inhibiting bone resorption [36]. Therefore, RANKL/OPG ratio is a major determinant for bone volume and health [3].

For efficient osteoclast differentiation from the earliest identifiable osteoclast precursors (colony forming unit-granulocyte macrophages, CFU-GM), macrophage colony stimulating factor (M-CSF, CSF-1) is required [38]. M-CSF is a homodimeric glycoprotein, expressed by mesenchymal cells, including MSCs [39], and binds to its specific tyrosine kinase receptor (c-fms/CSF-R), which is expressed on CFU-GM [38]. M-CSF is essential for inducing proliferation and survival of osteoclast precursors and osteoclasts [40]. Importantly, M-CSF promotes the expression of RANK on CFU-GM enabling them to respond to RANKL for further differentiation along the osteoclastic lineage [38].* Ex vivo*, recombinant RANKL along with M-CSF sufficiently induce osteoclast differentiation from osteoclastic progenitors in the absence of any supportive cells [41].

## 3. Expression of Skeletal RANKL and Its Regulation

RANKL is expressed in multiple tissues including skeletal muscles, immune organs, vascular tissues, and mammary glands, where it exerts a physiological or a pathological role depending on micro-environmental factors [5, 37]. During the process of physiological bone remodelling, RANKL, the main osteoclastic effector, is expressed in a membrane bound form on many mesenchymal cells including MSCs, osteoblasts, osteocytes, and chondrocytes [31, 42–46]. Which form predominates is a point of extensive research with controversial non-conclusive results [47–49]. During normal bone modeling/remodeling, expression of basal RANKL is activated in response to osteotropic factors such as 1*α*,25(OH)_2_D_3_, parathyroid hormone (PTH), prostaglandin E2 (PGE2), IL-1, leukemia inhibitory factor (LIF), and oncostatin M [32, 50]. To maintain normal bone remodeling, negative regulation for RANKL expression and/or signaling is required. In addition to OPG, the main negative regulator for RANKL signaling, myriad molecules such as estrogen, and immune related mediators including IL-4 [51], IL-13 [51], IL-10 [52], IL-18 [53], IFN-*γ* [54], and IFN-*β* [55] act as osteoprotective factors against excessive bone destruction. They act via different mechanisms like interfering with increased RANKL expression or signaling, upregulating OPG expression, or inducing osteoclast apoptosis [56].

Interestingly, some of the cytokines and growth factors, such as IL-7, IL-12, IL-23, IL-6, and transforming growth factor beta (TGF-*β*), have shown dual osteoclastogenic and anti-osteoclastogenic properties depending on pathophysiological state of bone* in vivo* [57].* In vitro*, this dual effect was shown to depend on the density and differentiation stage of the osteoclast population [58, 59]. During pathologic bone remodeling associated with infection, inflammation, or malignancy, infiltrating cells within a skeleton, such as activated T [34, 60] and B lymphocytes [61], inflamed synovial fibroblasts [62], osteoclasts [63], endothelial cells [64], and cancer cells [65, 66], play a role in enhanced RANKL expression via direct or paracrine mechanisms. These cells can directly express RANKL [61, 62, 65], which may be in a soluble form [67]. Moreover, these cells can enhance the expression of abnormal high levels of RANKL by mesenchymal cells via production of pro-osteoclastogenic factors and cytokines such as TNF, IL-17, IL-1, or epidermal growth factor (EGF) [68, 69].

## 4. Immunoregulatory Effects of MSCs

In addition to their stem/progenitor properties, MSCs display immunomodulatory functions and immunosuppressive actions both* in vitro* and* in vivo* [70]. The unique immunosuppressive feature of MSCs is especially beneficial in the treatment of autoimmune diseases, such as Crohn's disease [71], graft versus host disease (GVHD) [72, 73], and diabetes [74].

The immunosuppressive effects of MSCs are mediated either through MSCs-immune cells direct contact or by secretion of soluble factors [23, 75–77]. These factors include indoleamine 2,3-dioxygenase (IDO) or nitric oxide (NO) [78], IL-6 [79], IL-10 [80], and PGE2 [81], in addition to hemeoxygenase-1 [82], M-CSF [83], TGF-*β*1 [84], vascular endothelial growth factor (VEGF) [85], antagonistic variant of the chemokine CCL2 [86], TNF-stimulated gene-6 (TSG6) [87], interleukin 1 receptor antagonist (IL-1Ra) [88], soluble human leukocyte antigen-G5 (sHLA-G5) [89], and hepatocyte growth factor (HGF) [90, 91].

For the immunosuppressive action of MSCs to take place, a preliminary activation is elicited by proinflammatory cytokines released from the inflammatory microenvironment [70, 86, 92]. MSC activation can be induced either by IFN*γ* alone [93] or concomitant with TNF, IL-1*α*, or IL-1*β* [94–96]. In addition, the prominent inflammatory cytokine, IL-17, boosts immunosuppression by MSCs both* in vitro* and* in vivo* [97]. Importantly, MSCs show a dual regulatory role on immune cells where their action can be switched between immune stimulation and immune suppression according to the inflammatory milieu, and the levels and types of the inflammatory cytokines [70, 76, 98]. When the inflammation is mild, the effect of MSCs on immune cells is switched from immunosuppression to immune enhancing, drastically promoting the function of immune cells [76, 96, 98–101]. Insufficient inflammatory stimulus causes MSCs to enhance the immune response through the production of chemokines that recruit immune cells to sites of inflammation/injury. However, this low stimulus is not sufficient to allow the MSCs to exert their immune suppressive action on the immune cells, leading to accumulation of the latter and enhanced inflammation [96, 99]. This immune plasticity achieves a balance between proinflammatory and anti-inflammatory processes in order to maintain tissue integrity and homeostasis [98, 102].

Interestingly, administration of immunosuppressants was shown to disable the immunosuppressive action of MSCs, altering the therapeutic application of MSCs in immune-mediated disorders [98, 103].

Evidently, to achieve the anti-inflammatory therapeutic effect of MSCs for the treatment of inflammatory/autoimmune disorders, several factors should be considered. These factors include the dynamics of inflammation, the strength of immune system activation, the types of inflammatory cytokines, and the effects of immunosuppressant [98].

Under sufficient inflammatory conditions, MSCs exert their immune suppressive effects on the different types of immune cells through proliferation inhibition and functional modulation [77]. In addition, MSCs are able to suppress the differentiation of the immune cells from their precursors at the very early stages of the immune response. For example, human MSCs could exhibit an inhibitory effect on the differentiation of CD34^+^ progenitors and on monocyte differentiation into dendritic cells [83, 104, 105]. MSCs were shown to suppress the terminal differentiation of B cells into antibody secreting cells or plasma cells [106, 107]. Furthermore, MSCs were shown to suppress the differentiation of cytotoxic T lymphocytes from their precursors [108] and prevent the differentiation of naive CD4^+^ T cells into T helper 17 cells [109]. As osteoclasts are considered osteoimmune cells, under normal conditions or when the inflammatory status is not sufficient to elicit the immunosuppressive action, MSCs may normally support and enhance osteoclastogenesis. However, under sufficient inflammatory conditions, MSCs may suppress osteoclast formation. This hypothesis was supported by a number of* in vitro* human and murine studies in which osteoclast precursors were co-cultured with MSCs, and the effect of MSCs on* ex vivo* osteoclast formation and activity was assessed.

## 5. Studies Investigating the Effect of MSCs on Osteoclastogenesis* Ex Vivo*

Early studies identified that several preadipocytic, pre-osteoblastic stromal [110, 111], or mature osteoblasts [112] can support osteoclast formation when co-cultured with osteoclast precursors. Treatment of stromal cells/osteoblasts with a bone resorption stimulator like 1*α*,25(OH)_2_D_3_ or PTH and direct contact with osteoclast progenitors were essential for osteoclastogenesis. After discovery of RANKL, several* in vitro* and* in vivo* studies attempted to establish a link between the differentiation state of cells of osteoblastic lineage and their osteoclastogenesis supporting potential. However, the data were contradictory. Some of these studies showed that undifferentiated/immature osteoblastic/stromal cells exhibited not only stronger osteoclastogenesis supportive potential [39, 47, 113–115], but also higher RANKL expression [115]. In contrast, others concluded that commitment of osteoprogenitors into mature osteoblasts enhances their osteoclastogenesis supportive properties [116–119].

In one of the first studies to investigate the effect of MSCs on osteoclastogenesis [39], hBM-MSCs were co-cultured with the osteoclast progenitors, CD34^+^ hHSCs. MSCs supported the growth and differentiation of HSCs into functional osteoclasts in the absence of added hormones, cytokines, and growth factors. In this study, MSCs stimulated osteoclastogenesis in both cell contact and trans-well assays, indicating that both osteoclastogenic surface proteins and soluble factors mediated MSC action. However, in the trans-well assays, osteoclast formation was reduced by 75%. Therefore, it was concluded that cell-cell contact has a much greater potency in stimulating osteoclastogenesis than soluble mediators. By analyzing the effect of adding the osteotropic factor, 1*α*,25(OH)_2_D_3_, to the co-culture system, the authors reported that osteoclast formation was significantly enhanced in the presence of 10^−9^ M 1*α*,25(OH)_2_D_3_. However, the higher concentration (10^−8^ M) could not similarly enhance osteoclast formation [39]. Consistently, Baldock et al. [120] reported that maximal osteoclastogenic effect of 1*α*,25(OH)_2_D_3_ in co-cultures of osteoblasts and monocytes was at concentration of 10^−9^ M, while this effect was reduced at higher concentrations. Mbalaviele et al. [39] attributed the osteoclastogenic effect of MSCs to their expression of the well-known osteoclastogenic cytokines, IL-6, IL-11, M-CSF, stem cell factor, and LIF.

Recently, Ma et al. [121] demonstrated that the osteoclastogenesis-supportive role of MSCs is correlated with the inflammatory status of bone marrow from which they are derived. Bone marrow cells (osteoclast progenitors) from wild type mice were co-cultured with BM-MSCs derived from both wild type and systemic lupus erythematosus (SLE) model (MRL/lpr mice), in the presence of vitamin D3 and Prostaglandin E2. BM-MSCs isolated from MRL/lpr mice, characterized by chronic systemic inflammation and local bone marrow inflammation, showed enhanced osteoclastogenic activity compared to that of wild type MSCs.

The stimulatory action of MSCs on osteoclastogenesis represents only one aspect of their regulatory effect on osteoclast differentiation, whereas MSCs can also exert an inhibitory effect on this process. This dual effect of MSCs on differentiation and function of osteoclasts was clearly shown by Zhu et al. [122]. In this study, the authors firstly investigated the effect of non-treated MSCs on osteoclast development by co-culturing murine BM-MSCs with the murine osteoclast precursors, CD11b^+^ monocytes, in the absence or presence of relatively low doses of recombinant mouse M-CSF and RANKL. MSCs independently supported osteoclast development, and this effect was enhanced by M-CSF and RANKL. The stimulatory effect of non-treated MSCs on osteoclast formation was attributed to their ability to express the osteoclastogenic factors RANKL, M-CSF, and IL-6. It was supposed that addition of TNF, formerly known as TNF*α*, might strengthen the positive effect of MSCs on osteoclast formation, since TNF is one of the important proinflammatory cytokines that was shown to promote osteoclastogenesis [123–125]. However, the study reported that MSC treatment with TNF prior to culture with monocytes, or addition of TNF to the MSCs/monocytes co-culture system resulted in a strong inhibition of osteoclast formation and activity. TNF upregulated OPG expression by MSCs in a time- and dose-dependent manner, while it slightly downregulated M-CSF, RANKL, and IL-6 expression. Furthermore, when TNF-stimulated MSCs and monocytes were separated by a 0.4 *µ*m pore size membrane, the number of osteoclasts was increased indicating that not only soluble factors, but also surface proteins contributed to the inhibitory effect. Hence, TNF could switch the effect of MSCs on osteoclastogenesis from being supportive to being suppressive. This action of TNF may be considered a part of its role as a proinflammatory mediator enhancing the MSC immunosuppressive effects. MSC inhibition of inflammation associated osteolysis may be one of their unique immunosuppressive characteristics. Importantly, treatment of MSCs with rheumatoid arthritis synovial fluid (RASF), in which the concentrations of TNF were detected, modulated osteoclast generation in a close relation with the TNF level in RASF. MSCs promoted osteoclast formation when TNF concentration was relatively low, while they inhibited osteoclast generation after treatment with high TNF concentrations. These studies provided further evidence for the ability of MSCs to switch between the pro- and anti-inflammatory phenotypes [98]. It is noteworthy that the immunosuppressive effect of the MSCs was not always correlated with the dose of TNF in RASF in some patients. Therefore, it is possible that other factors in RASF may regulate MSC effect on osteoclastogenesis [122].

When studying the effect of MSCs on osteoclastogenesis, Oshita et al. [126] co-cultured MSCs with peripheral blood mononuclear cells (PBMCs), stimulated with relatively high levels of RANKL and M-CSF using a trans-well system. Under these conditions, MSCs exerted a suppressive effect on osteoclast differentiation and activity and this effect was partially attributed to OPG expression. It is suggested that these high levels of RANKL and M-CSF may have a similar effect as TNF stimulating the MSC anti-osteoclastogenic action. Unexpectedly, even in the absence of RANKL and M-CSF, MSCs constitutively produced OPG in levels sufficient to inhibit osteoclastogenesis. However, this latter finding was contradictory with that reported by Mbalaviele et al. [39] and Zhu et al. [122] and needs to be further tested.

Oshita et al. [126] proposed that not only OPG, but also other soluble mediators might be involved in inhibition of osteoclastogenesis by MSCs. The effect of MSCs on osteoclastogenesis and the involved mediators were also investigated by Takano et al. [127]. They reported that osteoclast formation was significantly inhibited in the presence of MSCs through secretion of the inhibitory factors OPG and IL-10. It is noteworthy that Oshita et al. [126] were unable to detect IL-10 in their culture system. IL-10 is an immunosuppressive and anti-inflammatory cytokine, which plays a critical role in limiting tissue injury during infections. It also has a role in protection against autoimmunity by limiting the duration and intensity of immune and inflammatory reactions. IL-10 is one of the immune regulatory cytokines secreted by MSCs as a part of their immunosuppressive reaction [128].* In vitro* and* in vivo* studies have shown an important role for IL-10 in suppressing osteoclastogenesis [52, 129]. IL-10 inhibits early stages of osteoclast differentiation through disrupting RANKL induced signaling [52] or co-stimulatory signals [130].

Takano et al. [127] also reported the secretion of TGF-*β*1 by MSCs in their co-culture system. The role of TGF-*β*1 in osteoclastogenesis and bone resorption is very complex and biphasic [131]. In culture, it depends on many factors including TGF-*β*1 concentration [57, 131]. TGF-*β*1 seems to stimulate osteoclast development [132], survival [133], and recruitment [134], mostly at low doses [131]. On the other hand, it inhibits osteoclastogenesis [132, 135] and promotes osteoclast apoptosis [136], particularly at high concentrations [131]. However, it is suggested that TGF-*β*1 effect on bone resorption* in vivo* depends on the local microenvironment such as the presence of other pro- or anti-osteoclastic cytokines [57].

Importantly, Takano et al. [127] did not add the commonly used RANKL and M-CSF to the culture medium; instead, their culture medium contained heat treated conditioned medium derived from the rat osteoblastic cell line ROS 17/2.8 (htROSCM). htROSCM strongly stimulated osteoclast differentiation in the presence of 1*α*,25(OH)_2_D_3_ in rat bone marrow culture systems [137–140]. However, when MSCs were added in the htROSCM-containing culture system, the outcome was reversed where osteoclast formation was inhibited [127]. Interestingly, the non-heat-treated ROSCM strongly inhibited osteoclast formation. Therefore, the stimulatory activity of htROSCM is apparently derived from heat stable protein(s), which are different from the heat labile colony stimulating factors (CSFs), including M-CSF and granulocyte macrophage CSF (GM-CSF). It is possible that the stimulatory effect of htROSCM is due to the denaturation of the inhibitory factor(s). Furthermore, heat treatment may activate some stimulating factor(s) [137]. Further characterization of htROSCM and the factor(s) responsible for their activity is still required. Takano et al. [127] showed that MSCs in the presence of htROSCM, a potent stimulator for osteoclastogenesis, exerted an inhibitory effect on osteoclast differentiation. In this study, the efficiency of MSCs to inhibit osteoclastogenesis was higher (by 10-fold) in the direct cell-cell contact co-culture system in comparison with the contact-free trans-well system, indicating that some putative potent anti-osteoclastogenic molecules are expressed on the MSCs. Another study by Varin et al. [141] investigated the effect of MSCs on osteoclast formation through the direct interaction of the MSC surface marker CD200 with its receptor (CD200R), expressed on the osteoclast precursors.

CD200 is a newly identified marker for MSCs and could be efficiently used to purify native MSCs [142]. CD200 is an immunoglobulin superfamily member expressed on various types of cells and acts as immunosuppressive cell surface glycoprotein [143]. CD200 receptor is a type 1 transmembrane glycoprotein, mainly expressed on cells of myeloid lineage such as monocytes and macrophages [141, 143]. The CD200-CD200R interaction could initiate an immunosuppressive signal that leads to different immunomodulatory actions and anti-inflammatory effects downregulating several immune cell functions, especially macrophages [143, 144].

**Figure 2:**
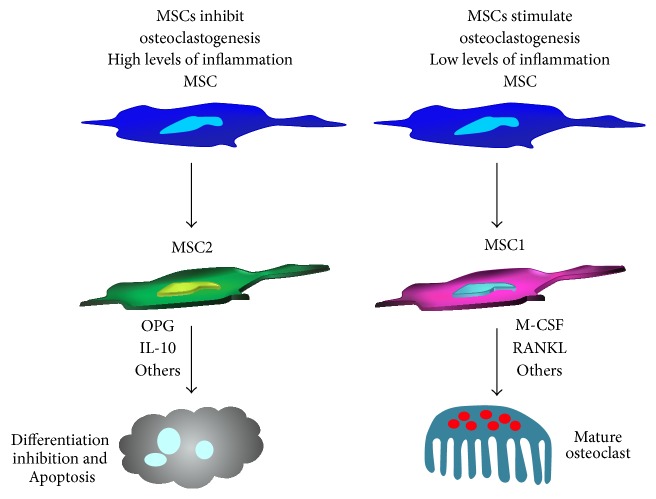
The dual effect of MSCs on osteoclastogenesis.

Varin et al. [141] demonstrated that CD200-CD200R interaction can block osteoclast formation and their bone degradation capacity by inhibiting the downstream RANK signaling pathway. CD200^+^, and not CD200^−^, MSC population significantly suppressed osteoclast formation. However, both populations expressed similar levels of OPG indicating that the inhibitory effect of CD200^+^ fraction is independent of OPG secretion. It is noteworthy that the total population of MSCs could exert an inhibitory effect on osteoclast differentiation. Importantly, the co-culture system in this study contained relatively high concentrations of the osteoclastogenic factors M-CSF and RANKL. The dual modulatory role of MSCs on osteoclastogenesis is shown in Figure 2.

It is noteworthy that the expression of CD200 on MSCs is origin dependent. Bone marrow is the most traditional source of CD200^+^ MSCs; however, its expression pattern was found to be varying from high to medium and low according to the donor. Meanwhile, umbilical cord blood derived MSCs (UCB-MSCs) were constantly negative for CD200 [143]. In addition, it was found that fetal, but not placental, MSCs preferentially express CD200 [145]. Similarly, CD200 was found to be a potential marker for visceral adipose stem cells (VS-ASC) but not subcutaneous adipose stem cells (SC-ASC) [146, 147]. Interestingly, MSCs isolated from Wharton's jelly expressed CD200 at higher proportions compared to bone marrow and adipose tissue MSCs, suggesting that the former could offer more immunomodulatory capacities [144].

The effect of the inflammatory signals on the MSC expression of CD200 was investigated, where neither Wharton's jelly nor adipose tissue MSCs showed a modulation of their CD200 expression upon inflammatory stimulation. In contrast, BM-MSCs showed an increase in the expression of CD200 when treated with proinflammatory cytokines. INF-*α*, TNF, and IL-1 induced a slight increase, while IFN*γ* induced a significant upregulation of CD200 expression on BM-MSCs [144]. Purified from an appropriate source, CD200^+^ MSC population may represent a potent transplantable therapeutic modality for application in several inflammatory and autoimmune diseases.

## 6. The* In Vivo* Anti-Osteoclastogenic Effect of MSCs

The therapeutic approach of MSC transplantation to recover bone loss in different models of inflammatory diseases associated with abnormal bone metabolism, such as primary or secondary osteoporosis and rheumatoid arthritis (RA), has been reported. MSC transplantation improved bone matrix formation and reduced bone resorption leading to improved bone density and structure in steroid induced osteoporosis model [148], rat model of adjuvant arthritis (AA) [127], and MRL/lpr mice (model of SLE with secondary osteoporosis) [121]. However, little was known about the therapeutic targets of MSC transplants in these models. Ma et al. [121] showed that BM-MSCs of MRL/lpr mice were the therapeutic targets of transplanted human MSCs derived from healthy donors. In both SLE patients and MRL/lpr mice, increased levels of the proinflammatory cytokine IL-17 in their bone marrow impaired the osteogenic potential and accelerated the osteoclastic inductive effect of BM-MSCs. hMSC transplantation led to downregulation for the abnormal expression of IL-17 and recovery of the impaired functions of recipient BM-MSCs, resetting the bone homoeostasis.

Importantly, in accordance with the* in vitro* results, there was no effect for MSC transplantation in wild type mice on bone metabolism, suggesting that recipient's inflammatory milieu might influence the transplanted MSCs' ability to correct the imbalanced bone metabolism [121].

## 7. Conclusion

Based on the aforementioned* in vitro* studies, it can be concluded that MSCs have a dual effect on osteoclasts, similar to their effect on other immune cells. This effect is dependent on the microenvironment. The osteoclastic modulatory effect of MSCs seems to be correlated with the intensity of the osteoclast induction conditions. In the studies that support the osteoclastogenic stimulatory role of MSCs, osteoclast inducing factor(s) such as M-CSF and RANKL were either absent [39, 121] or present at low concentrations [122]. However, in studies addressing the inhibitory effect of MSCs on osteoclastogenesis, a strong osteoclast inducing factor was found. In two of these studies, a relatively high concentration of the commonly used osteoclastogenic cytokines, RANKL and M-CSF, was added to the culture media, leading to stimulation of MSC anti-osteoclastogenic effect [126, 141]. However, treatment of MSCs with the proinflammatory cytokine TNF prior to co-culture with monocytes or addition of TNF to the MSCs/monocytes co-culture system switched the effect of MSCs on osteoclastogenesis from being supportive to being suppressive [122]. Moreover, when a potent osteoclast-inducing medium (htROSCM) was used, MSCs significantly suppressed osteoclastogenesis in this system [127] (Table 1). Hence, it can be postulated that the presence of intense osteoclast inducing factors in co-culture systems may create a state similar to the inflammatory pathological conditions* in vivo*, which stimulates MSCs to exhibit their osteoclastogenic suppressive effect. However, this speculation needs further investigation, which may be beneficial in guiding the future therapeutic use of MSCs in inflammatory bone loss disorders.

## 8. Future Perspectives

(i) The regulatory effect of MSCs on osteoclasts seems to be dual and dependent on the inflammatory/immune status of the microenvironment in which they are applied. As previously shown, exposing MSCs to TNF switched their action on osteoclasts from being supportive to being suppressive; therefore, the effect of different types and levels of other proinflammatory and/or pro-osteoclastogenic factors such as IL-1*α*, IL-1*β*, and IFN*γ* on the osteoclastogenic modulatory role of MSCs needs to be also evaluated. It is noteworthy that several cytokines including TGF-*β*1, IL-4, and IL-1*β* were found to upregulate OPG expression in marrow stromal cells ST2, osteoblastic cells MC3T3-E1 [149], and endothelial cells [150], and they may also be involved in upregulating the expression of OPG in MSCs.
(ii) Since MSC therapeutic efficacy in treatment of inflammatory conditions can be enhanced by precon-ditioning of MSCs with proinflammatory cytokines, growth factors, or small molecules [151], similarly, MSC pretreatment with these factors may also be recommended to promote their anti-osteoclastogenic action. On the other hand, systemic or local profiling of pro-osteoclastogenic cytokines in patients with inflammatory bone loss disorders before MSC administration may be beneficial in determining the appropriate disease stage/timing at which MSCs should be applied to exert the required anti-osteoclastogenic therapeutic effect.
(iii) In addition to CD200, surface proteins which participate in the modulatory role of MSCs on osteoclastogenesis need further investigation. This would specify MSC subpopulations with anti-osteoclastogenic potential that would have a promising clinical use in inflammatory/autoimmune bone loss diseases.
(iv) With advancing age, BM-MSCs showed decrease in proliferation rate, differentiation capacity, number of osteoprogenitor cells, and bone migration capacity [152, 153]. Whether aging would also affect the role of MSCs on osteoclast formation and activity requires to be investigated, taking into account that aging is characterized by increased inflammatory milieu [154].

## Figures and Tables

**Table 1:** Studies addressing the effects of MSCs on osteoclastogenic culture systems.

Reference	Source of cells	Osteoclast precursors	Supplemented factors which support osteoclastogenesis				Osteoclastogenesis related factors secreted by MSCs
			RANKL	M-CSF	1,25(OH)_2_D_3_	Others	
[39]	Human	CD34^+^ HSCs	—	—	None or 10^−9^ or 10^−8^ M		IL-6, IL-11, M-CSF, stem cell factor, and LIF
[121]	Mice	Bone marrow cells	—	—	+ve	Prostaglandin E2	—
[122]	Mice	CD11b^+^ monocytes	None or 10 or 20 ng/mL	10 or 20 ng/mL	—	TNF may be present	RANKL, M-CSF, IL-6, and OPG
[126]	Human	PBMCs	50 ng/mL	50 ng/mL	None or 10^−7^ M		OPG
[141]	Human	PBMCs	50 ng/mL	50 ng/mL	—		—
[127]	Rats	Bone marrow cells	—	—	10^−8^ M	htROSCM was added to the culture media	TGF-*β*1, OPG, and IL-10

## References

[1] Kini U., Nandeesh B. N., Fogelman I., Gnanasegaran G., Wall H. (2012). Physiology of bone formation, remodeling, and metabolism. *Radionuclide and Hybrid Bone Imaging*.

[2] Parfitt A. M. (2002). Targeted and nontargeted bone remodeling: relationship to basic multicellular unit origination and progression. *Bone*.

[3] Theoleyre S., Wittrant Y., Tat S. K., Fortun Y., Redini F., Heymann D. (2004). The molecular triad OPG/RANK/RANKL: involvement in the orchestration of pathophysiological bone remodeling. *Cytokine and Growth Factor Reviews*.

[4] Zelzer E., Olsen B. R. (2003). The genetic basis for skeletal diseases. *Nature*.

[5] Walsh M. C., Choi Y. (2014). Biology of the RANKL-RANK-OPG system in immunity, bone, and beyond. *Frontiers in Immunology*.

[6] Friedenstein A. J., Chailakhyan R. K., Latsinik N. V., Panasyuk A. F., Keiliss-Borok I. V. (1974). Stromal cells responsible for transferring the microenvironment of the hemopoietic tissues. Cloning in vitro and retransplantation in vivo. *Transplantation*.

[7] da Silva Meirelles L., Chagastelles P. C., Nardi N. B. (2006). Mesenchymal stem cells reside in virtually all post-natal organs and tissues. *Journal of Cell Science*.

[8] Guillot P. V., Gotherstrom C., Chan J., Kurata H., Fisk N. M. (2007). Human first-trimester fetal MSC express pluripotency markers and grow faster and have longer telomeres than adult MSC. *Stem Cells*.

[9] Cananzi M., Atala A., De Coppi P. (2009). Stem cells derived from amniotic fluid: new potentials in regenerative medicine. *Reproductive BioMedicine Online*.

[10] Bianco P., Robey P. G. (2015). Skeletal stem cells. *Development*.

[11] Pak J. (2011). Regeneration of human bones in hip osteonecrosis and human cartilage in knee osteoarthritis with autologous adipose-tissue-derived stem cells: a case series. *Journal of Medical Case Reports*.

[12] Wang L., Wang L., Cong X. (2013). Human umbilical cord mesenchymal stem cell therapy for patients with active rheumatoid arthritis: safety and efficacy. *Stem Cells and Development*.

[13] Vandermeulen M., Grégoire C., Briquet A., Lechanteur C., Beguin Y., Olivier D. (2014). Rationale for the potential use of mesenchymal stromal cells in liver transplantation. *World Journal of Gastroenterology*.

[14] Dominici M., Le Blanc K., Mueller I. (2006). Minimal criteria for defining multipotent mesenchymal stromal cells. The International Society for Cellular Therapy position statement. *Cytotherapy*.

[15] Ren G., Chen X., Dong F. (2012). Concise review: mesenchymal stem cells and translational medicine: Emerging issues. *Stem Cells Translational Medicine*.

[16] Kim N., Cho S.-G. (2013). Clinical applications of mesenchymal stem cells. *The Korean Journal of Internal Medicine*.

[17] Horwitz E. M., Gordon P. L., Koo W. K. K. (2002). Isolated allogeneic bone marrow-derived mesenchymal cells engraft and stimulate growth in children with osteogenesis imperfecta: implications for cell therapy of bone. *Proceedings of the National Academy of Sciences of the United States of America*.

[18] Carraro G., Perin L., Sedrakyan S. (2008). Human amniotic fluid stem cells can integrate and differentiate into epithelial lung lineages. *Stem Cells*.

[19] Jiang Y., Jahagirdar B. N., Reinhardt R. L. (2002). Pluripotency of mesenchymal stem cells derived from adult marrow. *Nature*.

[20] Prockop D. J., Kota D. J., Bazhanov N., Reger R. L. (2010). Evolving paradigms for repair of tissues by adult stem/progenitor cells (MSCs). *Journal of Cellular and Molecular Medicine*.

[21] Roddy G. W., Oh J. Y., Lee R. H. (2011). Action at a distance: systemically administered adult stem/progenitor cells (MSCs) reduce inflammatory damage to the cornea without engraftment and primarily by secretion of TNF-*α* stimulated gene/protein 6. *Stem Cells*.

[22] da Silva Meirelles L., Fontes A. M., Covas D. T., Caplan A. I. (2009). Mechanisms involved in the therapeutic properties of mesenchymal stem cells. *Cytokine & Growth Factor Reviews*.

[23] Madrigal M., Rao K. S., Riordan N. H. (2014). A review of therapeutic effects of mesenchymal stem cell secretions and induction of secretory modification by different culture methods. *Journal of Translational Medicine*.

[24] Väänänen H. K., Zhao H., Mulari M., Halleen J. M. (2000). The cell biology of osteoclast function. *Journal of Cell Science*.

[25] Roodman G. D. (2006). Regulation of osteoclast differentiation. *Annals of the New York Academy of Sciences*.

[26] Bar-Shavit Z. (2007). The osteoclast: a multinucleated, hematopoietic-origin, bone-resorbing osteoimmune cell. *Journal of Cellular Biochemistry*.

[27] Udagawa N., Takahashi N., Akatsu T. (1990). Origin of osteoclasts: mature monocytes and macrophages are capable of differentiating into osteoclasts under a suitable microenvironment prepared by bone marrow-derived stromal cells. *Proceedings of the National Academy of Sciences of the United States of America*.

[28] Endo-Munoz L., Evdokiou A., Saunders N. A. (2012). The role of osteoclasts and tumour-associated macrophages in osteosarcoma metastasis. *Biochimica et Biophysica Acta*.

[29] Boyce B., Yao Z., Xing L. (2009). Osteoclasts have multiple roles in bone in addition to bone resorption. *Critical Reviews in Eukaryotic Gene Expression*.

[30] Wong B. R., Josien R., Lee S. Y. (1997). TRANCE (tumor necrosis factor [TNF]-related activation-induced cytokine), a new TNF family member predominantly expressed in T cells, is a dendritic cell-specific survival factor. *Journal of Experimental Medicine*.

[31] Lacey D. L., Timms E., Tan H.-L. (1998). Osteoprotegerin ligand is a cytokine that regulates osteoclast differentiation and activation. *Cell*.

[32] Yasuda H., Shima N., Nakagawa N. (1998). Osteoclast differentiation factor is a ligand for osteoprotegerin/ osteoclastogenesis-inhibitory factor and is identical to TRANCE/RANKL. *Proceedings of the National Academy of Sciences of the United States of America*.

[33] Hsu H., Lacey D. L., Dunstan C. R. (1999). Tumor necrosis factor receptor family member RANK mediates osteoclast differentiation and activation induced by osteoprotegerin ligand. *Proceedings of the National Academy of Sciences of the United States of America*.

[34] Kung Y.-Y., Felge U., Sarosi I. (1999). Activated T cells regulate bone loss and joint destruction in adjuvant arthritis through osteoprotegerin ligand. *Nature*.

[35] Mellis D. J., Itzstein C., Helfrich M. H., Crockett J. C. (2011). The skeleton: a multi-functional complex organ: the role of key signalling pathways in osteoclast differentiation and in bone resorption. *Journal of Endocrinology*.

[36] Simonet W. S., Lacey D. L., Dunstan C. R. (1997). Osteoprotegerin: a novel secreted protein involved in the regulation of bone density. *Cell*.

[37] Kearns A. E., Khosla S., Kostenuik P. J. (2008). Receptor activator of nuclear factor kappaB ligand and osteoprotegerin regulation of bone remodeling in health and disease. *Endocrine Reviews*.

[38] Arai F., Miyamoto T., Ohneda O. (1999). Commitment and differentiation of osteoclast precursor cells by the sequential expression of c-Fms and receptor activator of nuclear factor kappaB (RANK) receptors. *The Journal of Experimental Medicine*.

[39] Mbalaviele G., Jaiswal N., Meng A., Cheng L., Van den Bos C., Thiede M. (1999). Human mesenchymal stem cells promote human osteoclast differentiation from CD34^+^ bone marrow hematopoietic progenitors. *Endocrinology*.

[40] Tanaka S., Miyazaki T., Fukuda A. (2006). Molecular mechanism of the life and death of the osteoclast. *Annals of the New York Academy of Sciences*.

[41] Quinn J. M. W., Elliott J., Gillespie M. T., Martin T. J. (1998). A combination of osteoclast differentiation factor and macrophage-colony stimulating factor is sufficient for both human and mouse osteoclast formation *in vitro*. *Endocrinology*.

[42] Kartsogiannis V., Zhou H., Horwood N. J. (1999). Localization of RANKL (Receptor activator of NF*κ*B ligand) mRNA and protein in skeletal and extraskeletal tissues. *Bone*.

[43] Silvestrini G., Ballanti P., Patacchioli F. (2005). Detection of osteoprotegerin (OPG) and its ligand (RANKL) mRNA and protein in femur and tibia of the rat. *Journal of Molecular Histology*.

[44] Carda C., Silvestrini G., Gomez de Ferraris M. E., Peydró A., Bonucci E. (2005). Osteoprotegerin (OPG) and RANKL expression and distribution in developing human craniomandibular joint. *Tissue and Cell*.

[45] Zhu W.-Q., Wang X., Wang X.-X., Wang Z.-Y. (2007). Temporal and spatial expression of osteoprotegerin and receptor activator of nuclear factor-*κ*B ligand during mandibular distraction in rats. *Journal of Cranio-Maxillo-Facial Surgery*.

[46] Nakashima T., Hayashi M., Fukunaga T. (2011). Evidence for osteocyte regulation of bone homeostasis through RANKL expression. *Nature Medicine*.

[47] Galli C., Fu Q., Wang W. (2009). Commitment to the osteoblast lineage is not required for RANKL gene expression. *The Journal of Biological Chemistry*.

[48] Xiong J., Onal M., Jilka R. L., Weinstein R. S., Manolagas S. C., O'Brien C. A. (2011). Matrix-embedded cells control osteoclast formation. *Nature Medicine*.

[49] Fumoto T., Takeshita S., Ito M., Ikeda K. (2014). Physiological functions of osteoblast lineage and T cell-derived RANKL in bone homeostasis. *Journal of Bone and Mineral Research*.

[50] Suda T., Takahashi N., Martin T. J. (1992). Modulation of osteoclast differentiation. *Endocrine Reviews*.

[51] Palmqvist P., Lundberg P., Persson E. (2006). Inhibition of hormone and cytokine-stimulated osteoclastogenesis and bone resorption by interleukin-4 and interleukin-13 is associated with increased osteoprotegerin and decreased RANKL and RANK in a STAT6-dependent pathway. *The Journal of Biological Chemistry*.

[52] Evans K. E., Fox S. W. (2007). Interleukin-10 inhibits osteoclastogenesis by reducing NFATc1 expression and preventing its translocation to the nucleus. *BMC Cell Biology*.

[53] Horwood N. J., Elliott J., Martin T. J., Gillespie M. T. (2001). IL-12 alone and in synergy with IL-18 inhibits osteoclast formation in vitro. *Journal of Immunology*.

[54] Ji J.-D., Park-Min K.-H., Shen Z. (2009). Inhibition of RANK expression and osteoclastogenesis by TLRs and IFNgamma in human osteoclast precursors. *The Journal of Immunology*.

[55] Takayanagi H., Kim S., Matsuo K. (2002). RANKL maintains bone homeostasis through c-fos-dependent induction of *interferon*-*β*. *Nature*.

[56] Zhao B., Ivashkiv L. B. (2011). Negative regulation of osteoclastogenesis and bone resorption by cytokines and transcriptional repressors. *Arthritis Research & Therapy*.

[57] Zupan J., Jeras M., Marc J. (2013). Osteoimmunology and the influence of pro-inflammatory cytokines on osteoclasts. *Biochemia Medica*.

[58] Janssens K., ten Dijke P., Janssens S., Van Hul W. (2005). Transforming growth factor-*β*1 to the bone. *Endocrine Reviews*.

[59] Yoshitake F., Itoh S., Narita H., Ishihara K., Ebisu S. (2008). Interleukin-6 directly inhibits osteoclast differentiation by suppressing receptor activator of NF-*κ*B signaling pathways. *The Journal of Biological Chemistry*.

[60] D'Amico L., Roato I. (2012). Cross-talk between T cells and osteoclasts in bone resorption. *BoneKEy Reports*.

[61] Onal M., Xiong J., Chen X. (2012). Receptor activator of nuclear factor *κ*B ligand (RANKL) protein expression by B lymphocytes contributes to ovariectomy-induced bone loss. *The Journal of Biological Chemistry*.

[62] Gravallese E. M., Manning C., Tsay A. (2000). Synovial tissue in rheumatoid arthritis is a source of osteoclast differentiation factor. *Arthritis and Rheumatism*.

[63] Boyce B. F., Xing L. (2008). Functions of RANKL/RANK/OPG in bone modeling and remodeling. *Archives of Biochemistry and Biophysics*.

[64] Okada T., Akikusa S., Okuno H., Kodaka M. (2003). Bone marrow metastatic myeloma cells promote osteoclastogenesis through RANKL on endothelial cells. *Clinical and Experimental Metastasis*.

[65] Huang L., Cheng Y. Y., Chow L. T. C., Zheng M. H., Kumta S. M. (2002). Tumour cells produce receptor activator of NF-kappaB ligand (RANKL) in skeletal metastases. *Journal of Clinical Pathology*.

[66] Wittrant Y., Théoleyre S., Chipoy C. (2004). RANKL/RANK/OPG: new therapeutic targets in bone tumours and associated osteolysis. *Biochimica et Biophysica Acta: Reviews on Cancer*.

[67] Zhang J., Dai J., Qi Y. (2001). Osteoprotegerin inhibits prostate cancer-induced osteoclastogenesis and prevents prostate tumor growth in the bone. *The Journal of Clinical Investigation*.

[68] Normanno N., De Luca A., Aldinucci D. (2005). Gefitinib inhibits the ability of human bone marrow stromal cells to induce osteoclast differentiation: implications for the pathogenesis and treatment of bone metastasis. *Endocrine-Related Cancer*.

[69] Zupan J., Komadina R., Marc J. (2012). The relationship between osteoclastogenic and anti-osteoclastogenic pro-inflammatory cytokines differs in human osteoporotic and osteoarthritic bone tissues. *Journal of Biomedical Science*.

[70] Ghannam S., Bouffi C., Djouad F., Jorgensen C., Noël D. (2010). Immunosuppression by mesenchymal stem cells: mechanisms and clinical applications. *Stem Cell Research & Therapy*.

[71] Dalal J., Gandy K., Domen J. (2012). Role of mesenchymal stem cell therapy in Crohn's disease. *Pediatric Research*.

[72] Le Blanc K., Rasmusson I., Sundberg B. (2004). Treatment of severe acute graft-versus-host disease with third party haploidentical mesenchymal stem cells. *The Lancet*.

[73] Le Blanc K., Frassoni F., Ball L. (2008). Mesenchymal stem cells for treatment of steroid-resistant, severe, acute graft-versus-host disease: a phase II study. *The Lancet*.

[74] Chhabra P., Brayman K. L. (2013). Stem cell therapy to cure type 1 diabetes: from hype to hope. *Stem Cells Translational Medicine*.

[75] Haddad R., Saldanha-Araujo F. (2014). Mechanisms of T-cell immunosuppression by mesenchymal stromal cells: what do we know so far?. *BioMed Research International*.

[76] Ma S., Xie N., Li W., Yuan B., Shi Y., Wang Y. (2014). Immunobiology of mesenchymal stem cells. *Cell Death and Differentiation*.

[77] Abumaree M., Al Jumah M., Pace R. A., Kalionis B. (2012). Immunosuppressive properties of mesenchymal stem cells. *Stem Cell Reviews and Reports*.

[78] Ren G., Su J., Zhang L. (2009). Species variation in the mechanisms of mesenchymal stem cell-mediated immunosuppression. *Stem Cells*.

[79] Djouad F., Charbonnier L.-M., Bouffi C. (2007). Mesenchymal stem cells inhibit the differentiation of dendritic cells through an interleukin-6-dependent mechanism. *STEM CELLS*.

[80] Batten P., Sarathchandra P., Antoniw J. W. (2006). Human mesenchymal stem cells induce T cell anergy and downregulate T cell allo-responses via the TH2 pathway: relevance to tissue engineering human heart valves. *Tissue Engineering*.

[81] Aggarwal S., Pittenger M. F. (2005). Human mesenchymal stem cells modulate allogeneic immune cell responses. *Blood*.

[82] Chabannes D., Hill M., Merieau E. (2007). A role for heme oxygenase-1 in the immunosuppressive effect of adult rat and human mesenchymal stem cells. *Blood*.

[83] Nauta A. J., Kruisselbrink A. B., Lurvink E., Willemze R., Fibbe W. E. (2006). Mesenchymal stem cells inhibit generation and function of both CD34^+^-derived and monocyte-derived dendritic cells. *Journal of Immunology*.

[84] Groh M. E., Maitra B., Szekely E., Koç O. N. (2005). Human mesenchymal stem cells require monocyte-mediated activation to suppress alloreactive T cells. *Experimental Hematology*.

[85] Kim Y.-S., Hong S.-W., Choi J.-P. (2009). Vascular endothelial growth factor is a key mediator in the development of T cell priming and its polarization to type 1 and type 17 T helper cells in the airways. *Journal of Immunology*.

[86] Rafei M., Campeau P. M., Aguilar-Mahecha A. (2009). Mesenchymal stromal cells ameliorate experimental autoimmune encephalomyelitis by inhibiting CD4 Th17 T cells in a CC chemokine ligand 2-dependent manner. *Journal of Immunology*.

[87] Lee R. H., Pulin A. A., Seo M. J. (2009). Intravenous hMSCs improve myocardial infarction in mice because cells embolized in lung are activated to secrete the anti-inflammatory protein TSG-6. *Cell Stem Cell*.

[88] Ortiz L. A., DuTreil M., Fattman C. (2007). Interleukin 1 receptor antagonist mediates the anti-inflammatory and anti-fibrotic effect of mesenchymal stem cells during lung injury. *Proceedings of the National Academy of Sciences of the United States of America*.

[89] Selmani Z., Naji A., Zidi I. (2008). Human leukocyte antigen-G5 secretion by human mesenchymal stem cells is required to suppress T lymphocyte and natural killer function and to induce CD4^+^CD25^high^FOXP3^+^ regulatory T cells. *Stem Cells*.

[90] Nicola M. D., Carlo-Stella C., Magni M. (2002). Human bone marrow stromal cells suppress T-lymphocyte proliferation induced by cellular or nonspecific mitogenic stimuli. *Blood*.

[91] Okunishi K., Dohi M., Fujio K. (2007). Hepatocyte growth factor significantly suppresses collagen-induced arthritis in mice. *Journal of Immunology*.

[92] Krampera M. (2011). Mesenchymal stromal cell ‘licensing’: a multistep process. *Leukemia*.

[93] Krampera M., Cosmi L., Angeli R. (2006). Role for interferon-*γ* in the immunomodulatory activity of human bone marrow mesenchymal stem cells. *Stem Cells*.

[94] van Buul G. M., Villafuertes E., Bos P. K. (2012). Mesenchymal stem cells secrete factors that inhibit inflammatory processes in short-term osteoarthritic synovium and cartilage explant culture. *Osteoarthritis and Cartilage*.

[95] Ren G., Zhang L., Zhao X. (2008). Mesenchymal stem cell-mediated immunosuppression occurs via concerted action of chemokines and nitric oxide. *Cell Stem Cell*.

[96] Li W., Ren G., Huang Y. (2012). Mesenchymal stem cells: a double-edged sword in regulating immune responses. *Cell Death & Differentiation*.

[97] Han X., Yang Q., Lin L. (2014). Interleukin-17 enhances immunosuppression by mesenchymal stem cells. *Cell Death & Differentiation*.

[98] Wang Y., Chen X. D., Cao W., Shi Y. (2014). Plasticity of mesenchymal stem cells in immunomodulation: pathological and therapeutic implications. *Nature Immunology*.

[99] Bernardo M. E., Fibbe W. E. (2013). Mesenchymal stromal cells: sensors and switchers of inflammation. *Cell Stem Cell*.

[100] Gazdic M., Volarevic V., Arsenijevic N., Stojkovic M. (2015). Mesenchymal stem cells: a friend or foe in immune-mediated diseases. *Stem Cell Reviews and Reports*.

[101] Renner P., Eggenhofer E., Rosenauer A. (2009). Mesenchymal stem cells require a sufficient, ongoing immune response to exert their immunosuppressive function. *Transplantation Proceedings*.

[102] Glenn J. D., Whartenby K. A. (2014). Mesenchymal stem cells: emerging mechanisms of immunomodulation and therapy. *World Journal of Stem Cells*.

[103] Chen X., Gan Y., Li W. (2014). The interaction between mesenchymal stem cells and steroids during inflammation. *Cell Death & Disease*.

[104] Jiang X.-X., Zhang Y., Liu B. (2005). Human mesenchymal stem cells inhibit differentiation and function of monocyte-derived dendritic cells. *Blood*.

[105] Ramasamy R., Fazekasova H., Lam E. W.-F., Soeiro I., Lombardi G., Dazzi F. (2007). Mesenchymal stem cells inhibit dendritic cell differentiation and function by preventing entry into the cell cycle. *Transplantation*.

[106] Corcione A., Benvenuto F., Ferretti E. (2006). Human mesenchymal stem cells modulate B-cell functions. *Blood*.

[107] Asari S., Itakura S., Ferreri K. (2009). Mesenchymal stem cells suppress B-cell terminal differentiation. *Experimental Hematology*.

[108] Rasmusson I., Ringdén O., Sundberg B., Le Blanc K. (2003). Mesenchymal stem cells inhibit the formation of cytotoxic T lymphocytes, but not activated cytotoxic T lymphocytes or natural killer cells. *Transplantation*.

[109] Duffy M. M., Pindjakova J., Hanley S. A. (2011). Mesenchymal stem cell inhibition of T-helper 17 cell- differentiation is triggered by cell-cell contact and mediated by prostaglandin E2 via the EP4 receptor. *European Journal of Immunology*.

[110] Udagawa N., Takahashi N., Akatsu T. (1989). The bone marrow-derived stromal cell lines MC3T3-G2/PA6 and ST2 support osteoclast-like cell differentiation in cocultures with mouse spleen cells. *Endocrinology*.

[111] Yamashita T., Asano K., Takahashi N. (1990). Cloning of an osteoblastic cell line involved in the formation of osteoclast-like cells. *Journal of Cellular Physiology*.

[112] Deyama Y., Takeyama S., Koshikawa M. (2000). Osteoblast maturation suppressed osteoclastogenesis in coculture with bone marrow cells. *Biochemical and Biophysical Research Communications*.

[113] De Grooth R., Kawilarang-de Haas E. W. M., van de Sande-Rijkers C. M. T., Nijweide P. J. (1998). The role of osteoblast density and endogenous interleukin-6 production in osteoclast formation from the hemopoietic stem cell line FDCP-mix C2GM in coculture with primary osteoblasts. *Calcified Tissue International*.

[114] Corral D. A., Amling M., Priemel M. (1998). Dissociation between bone resorption and bone formation in osteopenic transgenic mice. *Proceedings of the National Academy of Sciences of the United States of America*.

[115] Gori F., Hofbauer L. C., Dunstan C. R., Spelsberg T. C., Khosla S., Riggs B. L. (2000). The expression of osteoprotegerin and RANK ligand and the support of osteoclast formation by stromal-osteoblast lineage cells is developmentally regulated. *Endocrinology*.

[116] Otsuka E., Notoya M., Hagiwara H. (2003). Treatment of myoblastic C2C12 cells with BMP-2 stimulates vitamin D-induced formation of osteoclasts. *Calcified Tissue International*.

[117] Huang J. C., Sakata T., Pfleger L. L. (2004). PTH differentially regulates expression of RANKL and OPG. *Journal of Bone and Mineral Research*.

[118] Nakagawa K., Abukawa H., Shin M. Y., Terai H., Troulis M. J., Vacanti J. P. (2004). Osteoclastogenesis on tissue-engineered bone. *Tissue Engineering*.

[119] Ghosh-Choudhury N., Singha P. K., Woodruff K. (2006). Concerted action of Smad and CREB-Binding protein regulates bone morphogenetic protein-2-stimulated osteoblastic colony-stimulating factor-1 expression. *The Journal of Biological Chemistry*.

[120] Baldock P. A., Thomas G. P., Hodge J. M. (2006). Vitamin D action and regulation of bone remodeling: suppression of osteoclastogenesis by the mature osteoblast. *Journal of Bone and Mineral Research*.

[121] Ma L., Aijima R., Hoshino Y. (2015). Transplantation of mesenchymal stem cells ameliorates secondary osteoporosis through interleukin-17-impaired functions of recipient bone marrow mesenchymal stem cells in MRL/lpr mice. *Stem Cell Research & Therapy*.

[122] Zhu H., Jiang X.-X., Guo Z.-K. (2009). Tumor necrosis factor-*α* alters the modulatory effects of mesenchymal stem cells on osteoclast formation and function. *Stem Cells and Development*.

[123] Pfeilschifter J., Chenu C., Bird A., Mundy G. R., Roodman G. D. (1989). Interleukin-1 and tumor necrosis factor stimulate the formation of human osteoclastlike cells in vitro. *Journal of Bone and Mineral Research*.

[124] Steeve K. T., Marc P., Sandrine T., Dominique H., Yannick F. (2004). IL-6, RANKL, TNF-alpha/IL-1: interrelations in bone resorption pathophysiology. *Cytokine and Growth Factor Reviews*.

[125] Boyce B. F., Li P., Yao Z. (2005). TNF-alpha and pathologic bone resorption. *Keio Journal of Medicine*.

[126] Oshita K., Yamaoka K., Udagawa N. (2011). Human mesenchymal stem cells inhibit osteoclastogenesis through osteoprotegerin production. *Arthritis & Rheumatism*.

[127] Takano T., Li Y.-J., Kukita A. (2014). Mesenchymal stem cells markedly suppress inflammatory bone destruction in rats with adjuvant-induced arthritis. *Laboratory Investigation*.

[128] Shi Y., Hu G., Su J. (2010). Mesenchymal stem cells: a new strategy for immunosuppression and tissue repair. *Cell Research*.

[129] Al-Rasheed A., Scheerens H., Srivastava A. K., Rennick D. M., Tatakis D. N. (2004). Accelerated alveolar bone loss in mice lacking interleukin-10: late onset. *Journal of Periodontal Research*.

[130] Park-Min K.-H., Ji J.-D., Antoniv T. (2009). IL-10 suppresses calcium-mediated costimulation of receptor activator NF-kappa B signaling during human osteoclast differentiation by inhibiting TREM-2 expression. *Journal of Immunology*.

[131] Kasagi S., Chen W. (2013). TGF-beta1 on osteoimmunology and the bone component cells. *Cell & Bioscience*.

[132] Dieudonné S. C., Foo P., van Zoelen E. J. J., Burger E. H. (1991). Inhibiting and stimulating effects of TGF-beta 1 on osteoclastic bone resorption in fetal mouse bone organ cultures. *Journal of Bone and Mineral Research*.

[133] Fuller K., Lean J. M., Bayley K. E., Wani M. R., Chambers T. J. (2000). A role for TGF*β*
_1_ in osteoclast differentiation and survival. *Journal of Cell Science*.

[134] Pilkington M. F., Sims S. M., Dixon S. J. (2001). Transforming growth factor-*β* induces osteoclast ruffling and chemotaxis: potential role in osteoclast recruitment. *Journal of Bone and Mineral Research*.

[135] Chenu C., Pfeilschifter J., Mundy G. R., Roodman G. D. (1988). Transforming growth factor *β* inhibits formation of osteoclast-like cells in long-term human marrow cultures. *Proceedings of the National Academy of Sciences of the United States of America*.

[136] Hughes D. E., Dai A., Tiffee J. C., Li H. H., Munoy G. R., Boyce B. F. (1996). Estrogen promotes apoptosis of murine osteoclasts mediated by TGF-*β*. *Nature Medicine*.

[137] Kukita A., Kukita T., Hata K., Kurisu K., Kohashi O. (1993). Heat-treated osteoblastic cell (ROS17/2.8)-conditioned medium induces the formation of osteoclast-like cells. *Bone and Mineral*.

[138] Komine M., Kukita A., Kukita T., Ogata Y., Hotokebuchi T., Kohashi O. (2001). Tumor necrosis factor-*α* cooperates with receptor activator of nuclear factor *κ*B ligand in generation of osteoclasts in stromal cell-depleted rat bone marrow cell culture. *Bone*.

[139] Rahman M., Kukita A., Kukita T., Shobuike T., Nakamura T., Kohashi O. (2003). Two histone deacetylase inhibitors, trichostatin A and sodium butyrate, suppress differentiation into osteoclasts but not into macrophages. *Blood*.

[140] Nakamura T., Kukita T., Shobuike T. (2005). Inhibition of histone deacetylase suppresses osteoclastogenesis and bone destruction by inducing IFN-*β* production. *Journal of Immunology*.

[141] Varin A., Pontikoglou C., Labat E., Deschaseaux F., Sensebé L. (2013). CD200R/CD200 inhibits osteoclastogenesis: new mechanism of osteoclast control by mesenchymal stem cells in human. *PLoS ONE*.

[142] Delorme B., Ringe J., Gallay N. (2008). Specific plasma membrane protein phenotype of culture-amplified and native human bone marrow mesenchymal stem cells. *Blood*.

[143] Pietilä M., Lehtonen S., Tuovinen E. (2012). CD200 positive human mesenchymal stem cells suppress TNF-alpha secretion from CD200 receptor positive macrophage-like cells. *PLoS ONE*.

[144] Najar M., Raicevic G., Jebbawi F. (2012). Characterization and functionality of the CD200-CD200R system during mesenchymal stromal cell interactions with T-lymphocytes. *Immunology Letters*.

[145] Zhu Y., Yang Y., Zhang Y. (2014). Placental mesenchymal stem cells of fetal and maternal origins demonstrate different therapeutic potentials. *Stem Cell Research & Therapy*.

[146] Ong W. K., Tan C. S., Chan K. L. (2014). Identification of specific cell-surface markers of adipose-derived stem cells from subcutaneous and visceral fat depots. *Stem Cell Reports*.

[147] Mathis D. (2013). Immunological goings-on in visceral adipose tissue. *Cell Metabolism*.

[148] Lien C.-Y., Ho K. C.-Y., Lee O. K., Blunn G. W., Su Y. (2009). Restoration of bone mass and strength in glucocorticoid-treated mice by systemic transplantation of CXCR4 and Cbfa-1 Co-expressing mesenchymal stem cells. *Journal of Bone and Mineral Research*.

[149] Takai H., Kanematsu M., Yano K. (1998). Transforming growth factor-*β* stimulates the production of osteoprotegerin/osteoclastogenesis inhibitory factor by bone marrow stromal cells. *The Journal of Biological Chemistry*.

[150] Stein N. C., Kreutzmann C., Zimmermann S.-P. (2008). Interleukin-4 and interleukin-13 stimulate the osteoclast inhibitor osteoprotegerin by human endothelial cells through the STAT6 pathway. *Journal of Bone and Mineral Research*.

[151] Doorn J., Moll G., Le Blanc K., van Blitterswijk C., de Boer J. (2012). Therapeutic applications of mesenchymal stromal cells: paracrine effects and potential improvements. *Tissue Engineering B: Reviews*.

[152] Stolzing A., Jones E., McGonagle D., Scutt A. (2008). Age-related changes in human bone marrow-derived mesenchymal stem cells: consequences for cell therapies. *Mechanisms of Ageing and Development*.

[153] Zhou S., Greenberger J. S., Epperly M. W. (2008). Age-related intrinsic changes in human bone marrow derived mesenchymal stem cells and their differentiation to osteoblasts. *Aging Cell*.

[154] Jenny N. S. (2012). Inflammation in aging: cause, effect, or both?. *Discovery Medicine*.

